# Elevated fear states facilitate ventral hippocampal engagement of basolateral amygdala neuronal activity

**DOI:** 10.3389/fnbeh.2024.1347525

**Published:** 2024-02-14

**Authors:** Alexandra C. Ritger, Rachel K. Parker, Sydney Trask, Nicole C. Ferrara

**Affiliations:** ^1^Department of Neuroscience, Chicago Medical School, Rosalind Franklin University of Medicine and Science, North Chicago, IL, United States; ^2^Center for Neurobiology of Stress Resilience and Psychiatric Disorders, Rosalind Franklin University of Medicine and Science, North Chicago, IL, United States; ^3^Department of Psychological Sciences, Purdue University, West Lafayette, IN, United States; ^4^Purdue Institute for Integrative Neuroscience, Purdue University, West Lafayette, IN, United States; ^5^Department of Physiology and Biophysics, Chicago Medical School, Rosalind Franklin University of Medicine and Science, North Chicago, IL, United States

**Keywords:** basolateral amygdala, hippocampus, fear, memory, reconsolidation

## Abstract

Fear memory formation and retention rely on the activation of distributed neural circuits. The basolateral amygdala (BLA) and ventral hippocampus (VH) in particular are two regions that support contextual fear memory processes and share reciprocal connections. The VH → BLA pathway is critical for increases in fear after initial learning, in both fear renewal following extinction learning and during fear generalization. This raises the possibility that functional changes in VH projections to the BLA support increases in learned fear. In line with this, fear can also be increased with alterations to the original content of the memory via reconsolidation, as in fear elevation procedures. However, very little is known about the functional changes in the VH → BLA pathway supporting reconsolidation-related increases in fear. In this study, we used *in vivo* extracellular electrophysiology to examine the functional neuronal changes within the BLA and in the VH → BLA pathway as a result of fear elevation and standard fear retrieval procedures. Elevated fear expression was accompanied by higher BLA spontaneous firing compared to a standard fear retrieval condition. Across a range of stimulation frequencies, we also found that VH stimulation evoked higher BLA firing following fear elevation compared to standard retrieval. These results suggest that fear elevation is associated with an increased capacity of the VH to drive neuronal activity in the BLA, highlighting a potential circuit involved in strengthening existing fear memories.

## Introduction

Fear memories can be formed and retained as part of an adaptive process to promote survival (McGaugh, 2000). After initial formation, fear memories can be retrieved and modified as a means to incorporate new information into the original memory trace, referred to as reconsolidation (Misanin et al., 1968; Nader et al., 2000). Reconsolidation is a transient period of time following memory retrieval produced by brief re-exposure to stimuli or conditions present during initial learning (Nader et al., 2000; Hong et al., 2013). Importantly, new information during this re-exposure is required to trigger reconsolidation (Jarome et al., 2015; López et al., 2016; Ferrara et al., 2019b; Sinclair and Barense, 2019). These conditions render a memory labile, and thus provide an opportunity to alter the content of the memory (Duvarci and Nader, 2004; Kwapis et al., 2011). Studying the behavioral and neural processes involved in reconsolidation could be leveraged to understand how maladaptive, aberrant features of memories often seen in anxiety-based disorders, become exacerbated through subsequent subthreshold experiences (Careaga et al., 2016; Kida, 2019).

Pavlovian fear conditioning forms an associative memory with only a few pairings of an initially-neutral conditional stimulus (CS; e.g., a tone or context) and an aversive unconditional stimulus (UCS; e.g., a footshock). Following several CS-UCS pairings, when the CS is later presented alone during a retrieval or testing session, it elicits a conditional response (CR; Pavlov, 1927; Fanselow and Sterlace, 2014). A typical conditional response to a CS that signals footshock is freezing, defined as the absence of all movement except that required for respiration (Fanselow, 1980). Prior work has demonstrated that new information in the form of altered UCS intensity is sufficient to alter conditional responding in a manner that depends on the number of UCS presentations (Ferrara et al., 2019a; Popik et al., 2020, 2023; Bonanno et al., 2023). Here, a couple (two) pairings of a UCS at a reduced intensity can elevate freezing, whereas many (ten) can persistently deflate freezing (Ferrara et al., 2019a; Bonanno et al., 2023; see also Popik et al., 2020). These processes are believed to alter the original content of the memory via reconsolidation-like processes, and not necessarily create a new competing memory trace (e.g., Popik et al., 2020, 2023). Elevated fear provides a unique avenue to investigate the behavioral and neural underpinnings of reconsolidation, as it does not share behavioral similarities with other procedures that do not rely on reconsolidation, like extinction (Bouton, 2004; Duvarci and Nader, 2004). Instead, fear elevation procedures may take advantage of reconsolidation-like procedures as a result of learned fear that has not reached asymptote (Ozawa et al., 2017; Ferrara et al., 2019a). Here, an overlapping neural circuit may be recruited to support typical and elevated fear with an increase in functional drive.

The ventral hippocampus (VH) and basolateral region of the amygdala (BLA) both support contextual fear conditioning and directly project to one another (Zhang et al., 2001; Maren and Holt, 2004; Kishi et al., 2006; Cenquizca and Swanson, 2007; Twining et al., 2020). Excitatory CaMKII projections from the VH to the BLA contribute to time-dependent contextual fear generalization, and it has been suggested that neural circuits become reorganized to support increases in fear over time (Ortiz et al., 2019). Further, fear elevation increases BLA cellular activity, indexed with immediate early gene expression, and requires calcium impermeable AMPA receptor internalization (Ferrara et al., 2019a). Based on this, we hypothesized that the VH → BLA pathway may also functionally change to increase BLA neuronal activity following fear elevation.

The goal of the present study was to understand the functional neuronal changes within the BLA and in the VH → BLA pathway as a result of fear elevation. We replicate prior work, demonstrating elevated fear with two lower intensity UCS presentations 24 h following conditioning. We next used *in vivo* extracellular electrophysiology to quantify BLA firing and the impact of VH stimulation on BLA neuronal firing in standard retrieval and fear elevation groups. The present results contribute to literature examining reconsolidation-like processes and subsequent functional changes in the BLA evoked by the VH.

## Materials and methods

All experiments were approved by the Institutional Animal Care and Use Committee at Rosalind Franklin University of Medicine and Science.

### Subjects

Subjects were male Long Evans rats purchased from Envigo (*n* = 21; Indianapolis, IN, United States). The present study was a direct follow-up from a prior study and has included only male rats, though we recognize that this is a major limitation (Ferrara et al., 2019a). Rats were housed in standard rat cages (17 × 8.5 × 8″) and were all group housed (2–3/cage) in the Rosalind Franklin University animal facility with free access to food and water at all times and were maintained on a reverse light cycle (12/12 h light/dark). Adult rats arrived to the animal facility at postnatal day (PND) 64–69 and acclimated for at least 7 days prior to experimental procedures. Rats were randomly assigned to conditions, and all rats in a given cage were in the same treatment condition (e.g., all cage 1 rats were in the retrieval group, while all cage 2 rats were in the elevation group, etc.).

### Context fear conditioning, retrieval, and testing

**Figure 1:**
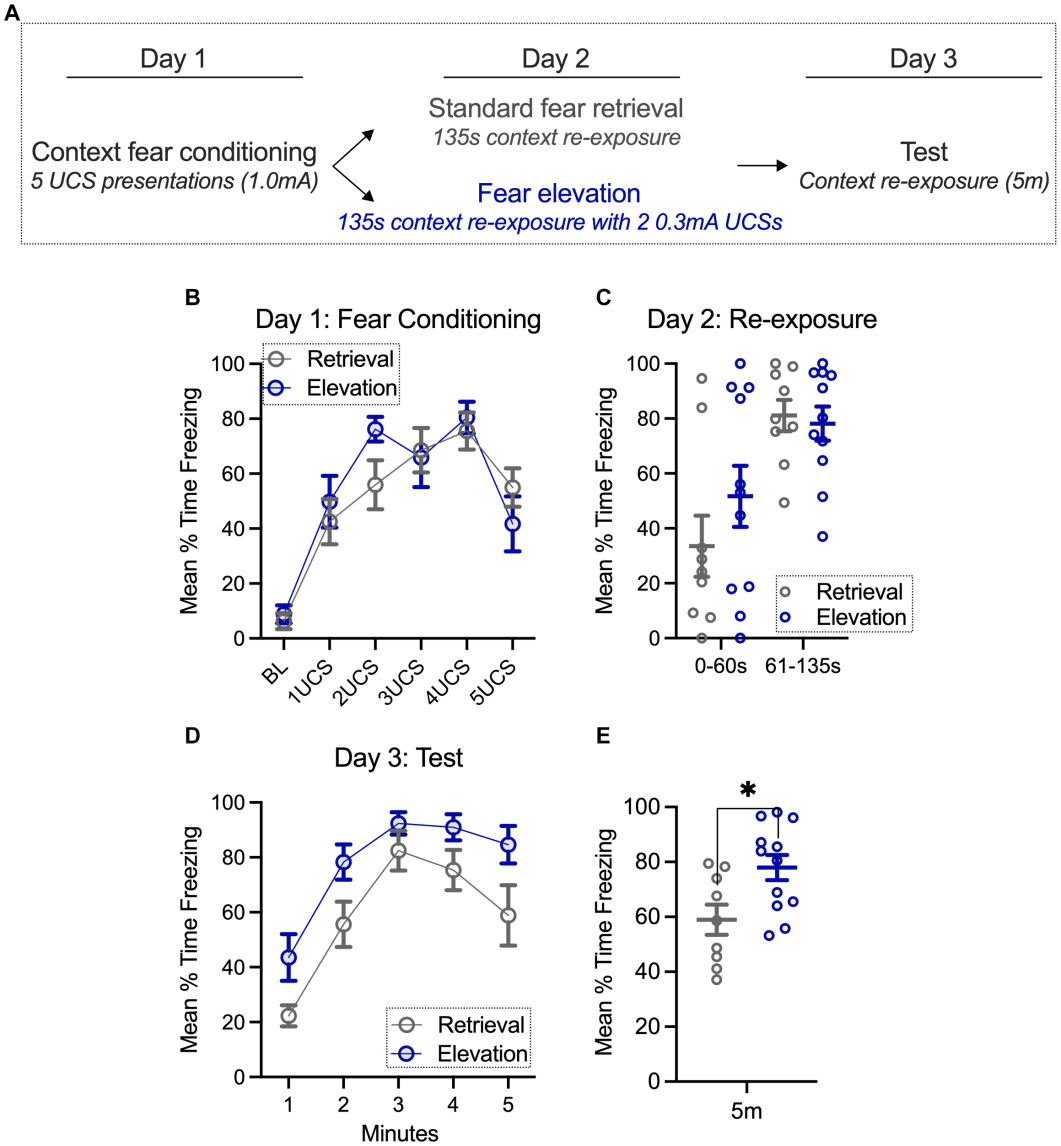
Fear can be elevated with additional mild footshock exposure 24 h after conditioning. **(A)** Groups were context fear conditioned, and the following day, exposed to standard retrieval or fear elevation conditions. On the final day, both groups were tested for fear to the context. **(B,C)** During context fear conditioning (day 1) and re-exposure (day 2), standard retrieval and fear elevation groups spent a similar amount of time freezing. **(D,E)** However, when testing for fear to the context (day 3), the fear elevation group spent significantly more time freezing than the standard retrieval group. Standard retrieval *n* = 9 rats; Fear elevation *n* = 12 rats. ^*^p < 0.05.

Rats were acclimated to transportation and handling from the homecage to the conditioning room for 3 days prior to conditioning. Chambers (Med Associates Inc., Fairfax, VT) for context fear conditioning contained a stainless steel grid floor to deliver footshock, were illuminated with a house light, and were cleaned with 1% acetic acid solution. Context fear conditioning consisted of a 140 s baseline, five 1.0 mA footshocks each separated by 60 s, and groups remained in the chamber for an additional 20 s (Figure 1A). The next day, the standard retrieval group was placed into the same chamber as conditioning for 135 s, while the fear elevation group received two additional 0.3 mA footshock exposures (60 s pre-footshock period, 60 s intershock interval, 15 s post period). Testing occurred 24 h later and consisted of identical chamber preparation as conditioning and standard retrieval/fear elevation days in the absence of footshock exposure. Freezing was defined as the cessation of movement excluding respiration and was automatically scored with VideoFreeze software (Med Associates) calibrated to a trained human observer.

### Anesthetized *in vivo* extracellular recordings

**Figure 2:**
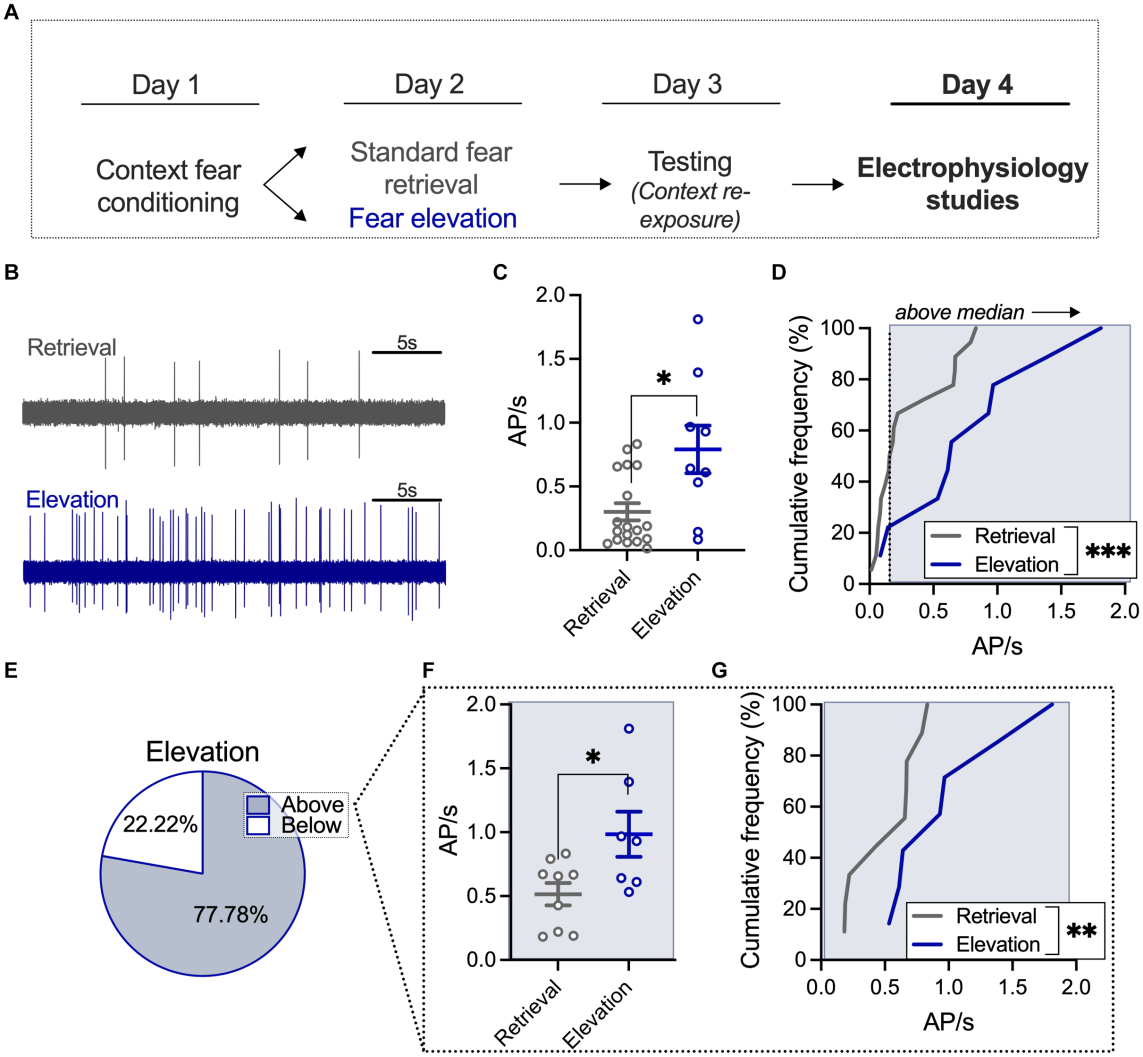
Elevated fear increases BLA neuronal firing frequency relative to a standard retrieval condition. **(A)** A subset of rats from the behavioral studies underwent electrophysiological recording 24 h after the behavioral testing session. **(B)** Sample traces of basolateral amygdala (BLA) firing after standard retrieval (top) or fear elevation (bottom). **(C)** Neurons in the fear elevation condition had significantly higher firing rates than neurons in the standard retrieval condition. **(D)** The distribution of firing rates was shifted towards higher firing rates in the fear elevation condition compared to the standard retrieval condition. **(E)** The median firing rate of the standard retrieval condition was used to split neurons into low and high firing groups. In the fear elevation condition, 78% of neurons fired faster the median rate and 22% fired slower the median rate. **(F)** Among neurons that fired faster than the median rate, the fear elevation condition had significantly higher firing rates than the standard retrieval condition, and **(G)** the distribution of firing rates was shifted towards higher firing rates in the fear elevation condition compared to the standard retrieval condition. Standard retrieval *n* = 4 rats (18 neurons); fear elevation *n* = 3 rats (9 neurons). ^*^p < 0.05, ^**^p < 0.01, and ^***^p < 0.001.

**Figure 3:**
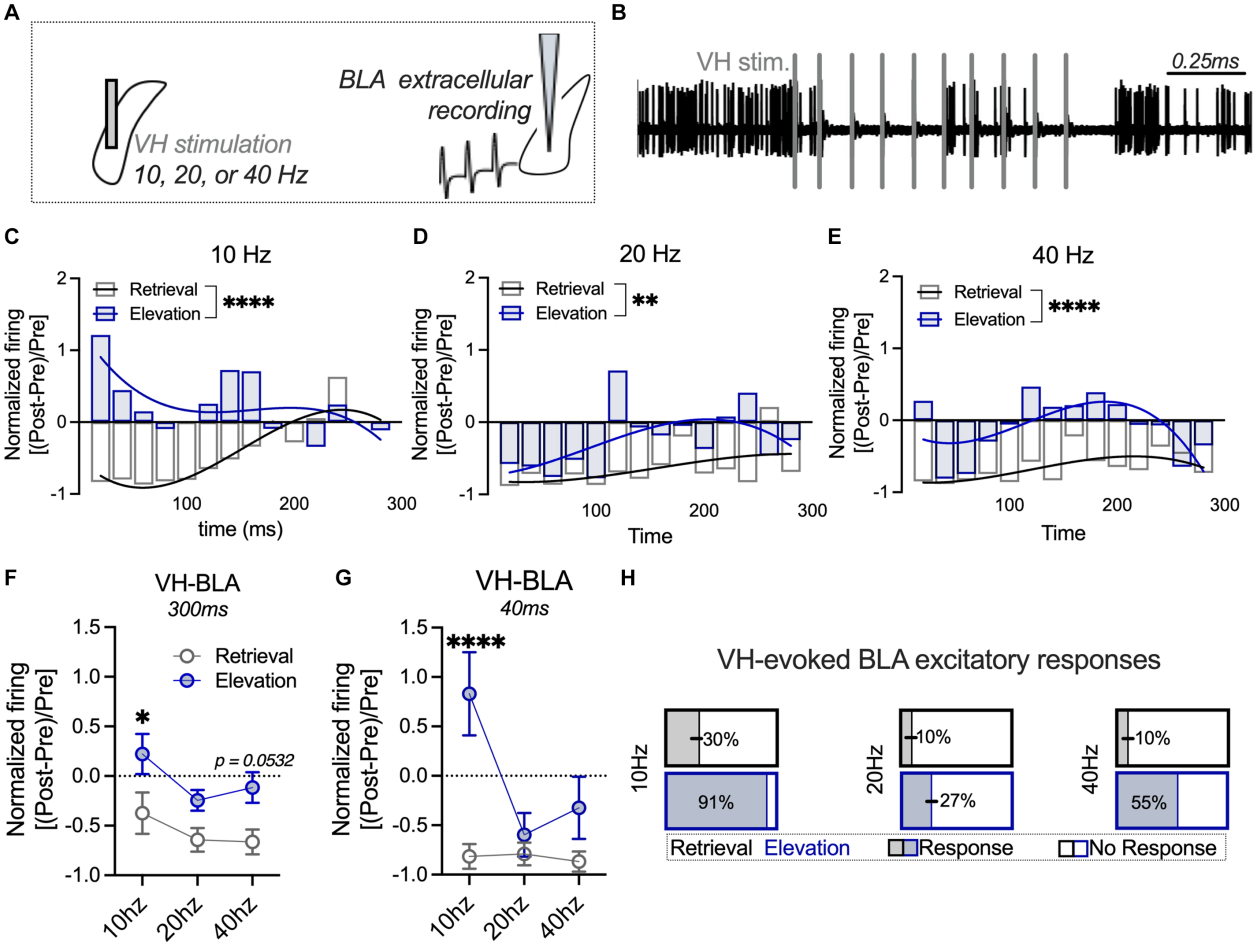
Elevated fear shifts VH-evoked BLA responses towards an excitatory profile relative to a standard retrieval condition. **(A)** The ventral hippocampus (VH) was stimulated at 10, 20, or 40 Hz, and extracellular firing was recorded in the basolateral amygdala (BLA). **(B)** Sample trace of BLA firing after VH stimulation (denoted with vertical gray bars). The BLA firing rate post-stimulation was normalized to the firing rate pre-stimulation, and a third-order polynomial was fit to the curve of the firing rate 0–300 ms post-stimulation in the standard retrieval and fear elevation conditions. **(C)** BLA neuronal firing was significantly different between fear elevation and standard retrieval conditions after VH 10 Hz stimulation, **(D)** 20 Hz stimulation, and **(E)** 40 Hz stimulation. **(F)** BLA activity 0–300 ms post-stimulation was averaged for each VH stimulation intensity (10, 20, or 40 Hz). Normalized BLA activity was significantly higher in the fear elevation condition than the standard retrieval condition after VH 10 Hz stimulation and modestly higher at 40 Hz stimulation. **(G)** In contrast, when only the first 0–40 ms were analyzed, VH 10 Hz stimulation evoked markedly higher normalized BLA activity in the fear elevation condition compared to the standard retrieval condition. **(H)** At VH 10 Hz stimulation, 91% of BLA neurons exhibited sustained excitatory responses in the fear elevation condition, compared to 30% of neurons in the standard retrieval condition. This pattern was consistent at 40 Hz (55% of fear elevation and 10% of standard retrieval BLA neurons), and weaker at 20 Hz (27% of fear elevation and 10% of standard retrieval BLA neurons). Standard retrieval *n* = 3 rats (10 neurons/frequency); fear elevation *n* = 3 rats (11 neurons/frequency). ^*^p < 0.05, ^**^p < 0.01 and ^****^p < 0.0001.

For electrophysiology experiments, a subset of rats from the behavioral results seen in Figure 1 were anesthetized with urethane (1.5 g/kg in 0.9% saline, injected intraperitoneally; i.p.) 24 h after the testing session on day 3. BLA neurons displayed in Figures 2, 3 were recorded within 4 h of when fully anesthetized. Rats were mounted onto a stereotaxic apparatus (Kopf Instruments, Tujunga, CA, United States) with body temperature maintained at 36–37°C (Model TC-1000, CWE, Inc., Ardmore, PA). A hole was drilled above the basolateral amygdala at A: −3.0 mm and L: −5.0 mm from bregma based on a rat brain atlas with varying ventral depth (Paxinos and Watson, 2006). A concentric bipolar stimulation electrode was lowered into the ventral hippocampus (Pl-NEX-100, Microprobes, Gaithersburg, MD, United States). Single-barrel glass recording electrodes were pulled using a vertical microelectrode puller (PE-2; Narishige, Tokyo, Japan) and broken for a 1–2 μm diameter tip. Electrodes were then filled with Pontamine [2% Chicago Sky Blue 6B (Sigma Aldrich, St. Louis, MO, United States) in 2 M NaCl]. The electrode was mounted onto the stereotaxic device and lowered with a hydraulic Microdrive (Frederick Haer & Co., Bowdoin, ME, United States). Extracellular signals were amplified with a headstage preamplifier (2400 Amplifier, Dagan Corporation, Minneapolis, MN, United States) connected to an amplifier (1800 Amplifier, A-M Systems, Sequim, WA, United States), and then to an audio monitor (Model AM8, Grass Instruments, West Warwick, RI, United States). Amplified signals were also digitized and recorded on a personal computer (Mac Pro/2.8, Apple, Cupertino, CA, United States) using Axograph X software (Sydney, Australia) and stored for off-line analysis.

Spontaneously firing neurons with at least a 3:1 signal:noise ratio were recorded at a low cut-off frequency of 100 Hz and high cut-off at 10 kHz (similar to Ritger et al., 2023). Neurons were recorded for a minimum of 5 min. The number of action potentials during the recording period were quantified and are displayed as a Hz value. Based on the median firing rate in the standard retrieval group, neurons that fired faster than 0.17 Hz over the course of the recording were considered “above” median firing, and neurons that fired slower than 0.17 Hz were considered “below” median firing.

The ventral hippocampus (VH) was stimulated at 0.5mA for train stimulation (10 pulses/train, Grass S88 stimulator, Grass Instruments). Firing frequency during 1 s baseline and 3 s post-stimulation was measured in 20 ms bins. Firing frequency was then normalized based on the peristimulus firing from each neuron, where post-stimulation BLA firing was normalized to the average baseline firing [(AP_Post_ − AP_Avg. Baseline_)/AP_Avg. Baseline_; Ferrara et al., 2023a]. Normalized responses were then averaged 300 ms and 40 ms following VH stimulation. Neurons were also divided based on response patterns. Here, an excitatory BLA “response” to VH stimulation was considered a normalized value of 0.45 or greater that was sustained for more than 40 ms within a 300 ms post stimulation period.

At the end of electrophysiology experiments, the 2% pontamine recording solution was injected into the brain to mark recording placement location. Rats were then rapidly decapitated and brains were removed and placed into 4% PFA for 24 h at 4°C. Brains were then transferred to 0.1 M PBS at 4°C until vibratome sectioning of tissue (60 microns) for placement reconstruction (please see Supplementary Figure S2).

### Statistical analyses

Graphpad PRISM software v10.0 (San Diego, CA, United States) was used for statistical analyses and figures. Data are represented as group averages with standard error of the mean (SEM) and, when indicated, cumulative frequency histograms. Behavioral and electrophysiology experiments were analyzed with unpaired *t*-tests (or Mann–Whitney *U* tests when the data were not normally distributed, based on a Kolmogorov–Smirnov test) and a 2-way ANOVA by conditions as appropriate. Sidak’s multiple comparisons post hocs were used to compare groups following significant main effects and interactions. Electrophysiology experiments were additionally analyzed using an *F* test to compare the global fit of a second- or third-order polynomial curve, as indicated.

## Results

### Fear can be elevated with additional mild footshock exposure 24 h after conditioning

Consistent with our prior work, rats were context fear conditioned with five 1.0 mA footshocks (Ferrara et al., 2019a). Using a repeated measures 2-way ANOVA (time, behavioral condition), we found a main effect of time (*F*_(5,95)_ = 28.21, *p* < 0.0001; Figures 1A,B), where freezing in both groups gradually increased with footshock exposure. There was not a main effect in the behavioral condition factor (standard retrieval, fear elevation; *F*_(1,19)_ = 0.210, *p* = 0.652) nor did we find a significant interaction (*F*_(5,93)_ = 1.385, *p* = 0.237), indicating that both groups similarly increased their freezing over time. On the second day of the behavioral experiment, rats were re-exposed to the conditioning chambers, where they either received two additional 0.3 mA footshock exposures or remained in the chamber for an equivalent amount of time (135 s, Figure 1C). Using the same repeated measures 2-way ANOVA as above, we found a main effect of time (*F*_(1,19)_ = 24.73, *p* < 0.0001), but no main effect of behavioral condition (*F*_(1,19)_ = 0.576, *p* = 0.457) and no interaction (*F*_(1,19)_ = 1.409, *p* = 0.250). These results confirm that both groups showed an increase from the first 60 s (prior to UCS presentations for elevation groups) and this did not differ between groups.

On the final day, groups were tested for fear to the context. Here, we used the same repeated measures 2-way ANOVA and found a main effect of time (*F*_(4,76)_ = 30.23, *p* < 0.0001) and a main effect of behavioral condition (*F*_(1,19)_ = 7.104, *p* = 0.0153) but no interaction (*F*_(4,76)_ = 0.647, *p* = 0.631; Figure 1D). These results suggest that while time did not differentially impact the groups, the elevation group froze more throughout the session. This was further confirmed by averaging freezing throughout the whole session; the fear elevation group froze more than the standard retrieval group (*t*_(19)_ = 2.665, *p* = 0.0153; Figure 1E). Among the standard retrieval and elevation groups, we noticed that a fraction of rats initially froze more during the re-exposure (day 2) than others. We were interested in whether freezing during testing (day 3) would be different in rats that we categorized on day 2 with less than 40% freezing (low), 41%–60% freezing (moderate), and 61%–100% freezing (high) during the first 60 s of the test, the time period prior to UCS exposure in the elevation group. Though underpowered for statistical analysis, there was no substantial difference in freezing in the elevation condition on the test day (day 3) with initially low, moderate, and high freezing. Surprisingly, rats in the standard retrieval group that froze more during the initial (0–60 s) period of the re-exposure (day 2), tended to have lower freezing during testing (day 3) relative to those that had higher initial freezing during the re-exposure (Supplementary Figure S1). This is consistent with overexpectation literature, where the anticipation of a reinforcer governs behavioral responding, and behavioral responding may decline more rapidly with higher anticipation (Lattal and Nakajima, 1998). Thus, the degree of freezing during re-exposure minimally impacts freezing during testing for elevation groups, but may be a factor contributing to variance in freezing among standard retrieval groups.

### Elevated fear increases BLA neuronal firing frequency relative to a standard retrieval condition

We next wanted to determine the impact of fear elevation on BLA neuronal activity, using *in vivo* extracellular recordings to capture spontaneously firing BLA neurons (Figures 2A,B; Supplementary Figure S2 for histological verification of placements). We used a *t*-test to quantify differences in neuronal firing in standard retrieval and fear elevation conditions and found that BLA firing is increased in the fear elevation group relative to the standard retrieval group (*U* = 39, *p* = 0.0308; Figure 2C). We next compared the distribution of BLA neuronal responses using a second-order polynomial curve fitted to each group using an *F* test and found that fear elevation significantly shifted response distribution such that a greater proportion of neurons displayed higher firing frequency relative to the standard retrieval group (*F*_(3,21)_ = 24.63, *p* < 0.0001; Figure 2D). We next investigated neurons in standard retrieval and fear elevation groups with firing frequency greater than the standard retrieval median (0.17 Hz). Among the fear elevation group, 77.78% of neurons fell into the “above median” category (Figure 2E), and firing among this population of neurons was faster than that of the standard retrieval group (*t*_(14)_ = 2.545, *p* = 0.0233; Figure 2F). We similarly found neuronal response distribution was shifted towards faster firing in the fear elevation relative to the standard retrieval group using an *F* test to compare the fit of a second-order polynomial curve (*F*_(3,10)_ = 18.81, *p* = 0.0002; Figure 2G). These results suggest BLA excitation corresponds to fear expression, where elevated fear expression may recruit BLA neuronal activity.

### Elevated fear shifts VH-evoked BLA responses towards an excitatory profile relative to a standard retrieval condition

VH inputs can excite or suppress the BLA, and this pathway is activated following fear memory formation (Hübner et al., 2014; Kim and Cho, 2020). Oscillations between the VH and BLA change during learned fear, where theta coupling (10 Hz) between the VH and BLA increases during memory consolidation and reconsolidation (Seidenbecher et al., 2003; Narayanan et al., 2007). It has been suggested that oscillations between the VH and BLA can influence synaptic plasticity as well as action potential frequency and synchronization (Terada et al., 2013; Totty and Maren, 2022). Oscillations between brain regions can engage excitatory principal neurons (10 Hz) or GABAergic neurons to suppress principal neurons (40 Hz). Based on this, we next investigated the impact of VH stimulation at a range of physiologically-relevant frequencies on BLA firing frequency in standard retrieval and fear elevation groups (Figures 3A,B). To initially investigate changes in BLA firing with VH stimulation, we examined neuronal firing normalized to the baseline (pre-stimulation) period over time beginning immediately following VH stimulation to 300 ms after stimulation. Temporal-related patterns in neuronal firing between the standard retrieval and fear elevation groups as a result of VH stimulation were compared across time by fitting a third-order polynomial curve to each data set. BLA neuronal firing was different between fear elevation and standard retrieval conditions after VH 10 Hz stimulation (*F*_(4,286)_ = 6.798; *p* < 0.0001; Figure 3C), 20 Hz (*F*_(4,286)_ = 3.5548, *p* = 0.0076; Figure 3D), and at 40 Hz (*F*_(4,286)_ = 7.121, *p* < 0.0001; Figure 3E), such that fear elevation conditions typically showed greater excitation and less inhibition relative to the standard retrieval group. We next averaged activity during the entire 300-ms period and compared averaged activity using a repeated measures 2-way ANOVA with stimulation frequency (10 Hz, 20 Hz, and 40 Hz) and behavioral condition (standard retrieval, fear elevation) as factors (Figure 3F). We found a main effect of behavioral condition (*F*_(1,19)_ = 12.95, *p* = 0.0019) and a main effect of stimulation frequency (*F*_(2,38)_ = 3.524, *p* = 0.0395) but there was no interaction (*F*_(2,38)_ = 1.360, *p* = 0.2051). *Post hoc* comparisons showed fear elevation had significantly higher normalized firing relative to the standard retrieval condition after VH at 10 Hz (*p* = 0.0301) and modestly at 40 Hz stimulation (*p* = 0.0532), but not at 20 Hz (*p* = 0.2246).

Based on the normalized responses over time (Figures 3C–E), we noticed a strong increase in BLA firing immediately following VH stimulation at 10 Hz that changed in a frequency-dependent manner. We next averaged normalized BLA firing in the first 40 ms after VH stimulation to capture the immediate, transient effects of VH engagement of BLA activity. Using the same repeated measures 2-way ANOVA as above, we found a main effect of behavioral condition (*F*_(1,19)_ = 8.243, *p* = 0.0098), a main effect of stimulation frequency (*F*_(2,38)_ = 6.961, *p* = 0.0027), and an interaction (*F*_(2,39)_ = 6.955, *p* = 0.0027; Figure 3G). *Post hoc* comparisons showed BLA firing was significantly greater in the fear elevation condition relative to the standard retrieval condition at 10 Hz (*p* < 0.0001), but there was no difference in VH-evoked BLA neuronal activity at 20 Hz (*p* = 0.9328) or at 40 Hz (*p* = 0.3645). Several neurons in the fear elevation condition also displayed sustained excitatory responses to VH stimulation. We next categorized neurons based on these response profiles and found that 10 Hz stimulation evoked the most sustained excitatory BLA responses (91%) relative to only 30% of neurons in the standard retrieval condition (Figure 3H). This pattern was consistent across frequencies, with 55% of BLA neurons displaying sustained excitatory responses in the fear elevation condition and 10% of neurons in the standard retrieval condition following 40 Hz stimulation, and the weakest difference at 20 Hz (27% of fear elevation and 10% of standard retrieval BLA neurons). These results collectively suggest that VH engagement of BLA excitatory activity increases in elevated fear states, with greater sustained responses after 40 Hz VH stimulation and greater immediate VH-evoked BLA activation at 10 Hz relative to the standard retrieval condition.

## Discussion

Here, we replicated our prior work (Ferrara et al., 2019a) that found that 2 weak shocks following contextual fear conditioning produces elevated contextual fear relative to a group that received standard memory retrieval. This fear elevation corresponded with increased BLA neuronal firing, complementing prior work that has demonstrated fear deflation corresponds with reduced neural activity in the BLA (Bonanno et al., 2023). Additionally, increases in BLA neuronal firing may be further facilitated by VH stimulation in a frequency-specific manner in the elevated fear condition. Together, these results suggest that fear elevation corresponds with an increased capacity of the VH to drive neuronal activity in the BLA. This work adds to a growing literature aiming to examine how subsequent experience with unconditional stimuli can modify conditional fear and provides a critical step forward in determining the circuit-level changes that occur with persistently elevated fear.

The VH and BLA have been extensively studied for contextual fear, including the formation, extinction, and return of fear after extinction. Notably, fear memory formation and renewal following extinction are dependent on an increase in activity in the VH → BLA pathway, linking increases in excitation between these two brain regions with increased fear expression (Orsini et al., 2011; Zhu et al., 2014; Kim and Cho, 2020; Liu et al., 2022; Brockway et al., 2023). Neural oscillations between these two brain regions are believed to play an important role in synaptic plasticity and neuronal firing can become phase locked to synchronized field potentials (Huerta and Lisman, 1993, 1995; Traub et al., 1998; Lesting et al., 2013; Kuga et al., 2022). A range of oscillatory frequencies have been implicated in memory, with an emphasis on theta and gamma range oscillations (Courtin et al., 2014; Stujenske et al., 2014; Castegnetti et al., 2021; Totty and Maren, 2022). Here, we stimulated the VH at physiologically relevant frequencies (10 Hz–40 Hz) to understand the impact on BLA neuronal firing in standard retrieval and fear elevation groups. We found that stimulation of the VH regardless of frequency evoked a greater degree of BLA excitatory responses in the fear elevation relative to the standard retrieval condition. Specifically, 20 and 40Hz VH stimulation frequencies had similar effects on BLA firing in both standard retrieval and fear elevation groups, though this tended to be higher in the fear elevation condition. However, we noticed strong, transient increases in BLA firing in the fear elevation condition immediately following VH 10 Hz stimulation, together suggesting frequency-specific VH-evoked alterations in BLA neuronal activity that depends on degree of fear. In line with this, theta range oscillations (including 10 Hz) change in a coordinated manner with fear expression and alter synaptic strength, where theta frequency from hippocampal inputs can increase BLA firing (Castegnetti et al., 2021). This may contribute to the increase in evoked firing of BLA neurons by 10 Hz VH stimulation in groups with elevated fear.

The BLA contains a diversity of inhibitory GABAergic interneurons that play an important role in balancing excitation and inhibition within the BLA for learned fear. Gamma oscillations engage inhibitory neuronal activity to guide precise firing patterns, and BLA gamma oscillations play an important role in fear memory retention and expression (Buzsáki and Wang, 2012; Courtin et al., 2014; Stujenske et al., 2014). Consistent with this, we found that 40 Hz VH stimulation (gamma range) increased BLA firing to a greater extent in the fear elevation relative to the standard retrieval condition, which may be similarly linked with fear memory retention and expression. Interestingly, BLA gamma power is influenced by parvalbumin GABAergic interneurons specifically, providing a potential source through which BLA principal neurons may be influenced with 40 Hz VH stimulation (Fu et al., 2022).

While one explanation for our behavioral results may be that elevated contextual fear is a result of additional context-shock pairings, our prior work (Ferrara et al., 2019a; Bonanno et al., 2023) and others (Popik et al., 2020, 2023) has demonstrated that, somewhat paradoxically, presenting even more context-weak shock pairings (e.g., ten) results in a decrease in freezing. Further, weak shock presentations alone are not enough to condition a robust fear memory themselves (Bonanno et al., 2023). Together, this suggests that weak shock presentations following initial learning do not sustain a fear memory alone, and instead require prior conditioning to subsequently alter fear expression in a bidirectional manner based on the number of UCSs presented. Further, fear elevation was dependent on the internalization of calcium impermeable AMPA receptor endocytosis (Ferrara et al., 2019a), a mechanism not required for initial learning (Rao-Ruiz et al., 2011; Hong et al., 2013). Thus, the present results are therefore unlikely a result of extended fear conditioning and instead rely on reconsolidation-like processing to persistently elevate fear.

Additionally, the present results share some similarities with stress-enhanced fear learning (Poulos et al., 2015; Perusini et al., 2016; Hassien et al., 2020), where prior experience with many inescapable footshocks exacerbates subsequent fear learning. This outcome is likely not the case here as, again, presenting several weak shocks actually reduces conditional freezing. Instead, we have recently argued that the behavioral difference between presenting a couple (e.g., two) or several (e.g., ten) weak shocks is similar to the difference between presentations of a few or several CSs following fear conditioning (Ferrara et al., 2023b). That is, presenting few CSs without unconditional stimuli will engage memory retrieval and reconsolidation processes whereas presenting many CSs will instead engage extinction processes, which creates a new inhibitory memory that competes with the original for expression. This interpretation and incorporation of prior work also suggests that our results are not a result of a sensitization-like hypothesis. In our prior work, ten UCS presentations does not increase later contextual fear as would be expected through a sensitization or contextual fear conditioning explanation, animals instead show reduced freezing (Ferrara et al., 2019a,b; Bonanno et al., 2023). This was true whether or not the weak shocks were presented in the same context as conditioning or a novel context (Bonanno et al., 2023). Further, the increases in fear mediated by presenting two additional shocks (as was the case in the present experiment) do not extend to novel contexts but do require GluA2 endocytosis, suggesting this elevation functions through retrieval-mediated memory updating (Ferrara et al., 2019a,b).

Another alternative explanation is that the animals are encoding a new representation of the weak shock that is distinct from the strong shock used to initially fear condition. In prior work, we have demonstrated that the weak shock itself does not produce a robust fear memory when used in the absence of the strong shock (Bonanno et al., 2023), suggesting that little aversive learning occurs at this amplitude.

Future work will test the necessity of the VH → BLA pathway both during the weak shock exposure as well as during testing, with the hypothesis that activity in this pathway will be necessary both during the weak shock exposure as well as during testing for fear elevation. Further, pathway stimulation during a standard memory retrieval should also produce behavior reflective of elevated freezing. Finally, while prior work suggests that changes in shock intensity between phases do not have sex-specific impacts on behavior in a modified version of this task (Bonanno et al., 2023), it is important for future work to systematically study how fear elevation impacts both males and females. Ongoing work dissecting this phenomenon using both sexes to determine if this phenomenon is robust across sex is critical to verify in the present paradigm and would greatly benefit the field, as distinct molecular mechanisms may support similar behavioral profiles across sex. This is especially true when considering that in humans, women are at increased risk for the development of anxiety disorders.

## Data availability statement

The raw data supporting the conclusions of this article will be made available by the authors, without undue reservation.

## Ethics statement

The animal study was approved by Rosalind Franklin University IACUC committee. The study was conducted in accordance with the local legislation and institutional requirements.

## Author contributions

AR: Formal analysis, Software, Visualization, Writing – original draft, Writing – review & editing. RP: Writing – original draft, Writing – review & editing. ST: Visualization, Writing – original draft, Writing – review & editing. NF: Conceptualization, Data curation, Formal analysis, Funding acquisition, Investigation, Methodology, Resources, Software, Supervision, Visualization, Writing – original draft, Writing – review & editing.

## Supplementary material

SUPPLEMENTARY FIGURE 1 Rats were categorized based on freezing responses during the first 60s of the re-exposure on Day 2; a time period prior to shock exposure in elevation groups. During testing (Day 3), freezing in the elevation group was relatively stable across ‘low’ (less than 40% freezing), ‘moderate’ (40%-60% freezing), and ‘high’ (greater than 61% freezing) freezing groups. However, rats that initially froze more during re-exposure (Day 2) in the standard retrieval group froze less during testing (Day 3).

SUPPLEMENTARY FIGURE 2 Histological verification of recording sites in the basolateral amygdala (BLA) and stimulation sites in the ventral hippocampus (VH). For BLA recording sites, dots represent the lowest possible point recorded in the BLA and dashed lines above dots include the area in which additional neurons in the track were quantified within the BLA.
