# Supplementary figures and images for: Elevated fear states facilitate ventral hippocampal engagement of basolateral amygdala neuronal activity
Source: Front Behav Neurosci. 2024 Feb 14;18:1347525. doi: 10.3389/fnbeh.2024.1347525 (PMC10899678; doi:10.3389/fnbeh.2024.1347525)

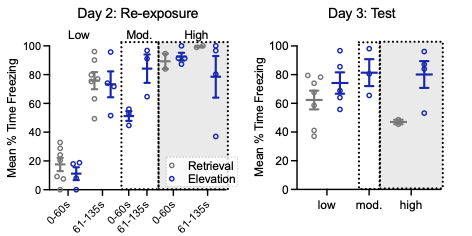

Supplement: SUPPLEMENTARY FIGURE 1 — Rats were categorized based on freezing responses during the first 60s of the re-exposure on Day 2; a time period prior to shock exposure in elevation groups. During testing (Day 3), freezing in the elevation group was relatively stable across ‘low’ (less than 40% freezing), ‘moderate’ (40%-60% freezing), and ‘high’ (greater than 61% freezing) freezing groups. However, rats that initially froze more during re-exposure (Day 2) in the standard retrieval group froze less during testing (Day 3). [file Image_1.tiff]

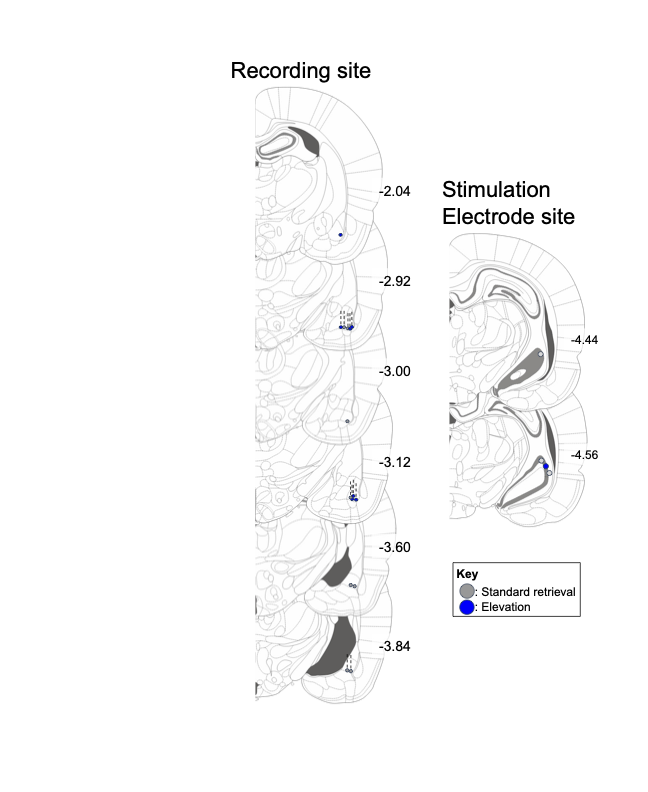

Supplement: SUPPLEMENTARY FIGURE 2 — Histological verification of recording sites in the basolateral amygdala (BLA) and stimulation sites in the ventral hippocampus (VH). For BLA recording sites, dots represent the lowest possible point recorded in the BLA and dashed lines above dots include the area in which additional neurons in the track were quantified within the BLA. [file Image_2.tiff]
